# Quantitative assessment of background parenchymal enhancement in breast MRI predicts response to risk-reducing salpingo-oophorectomy: preliminary evaluation in a cohort of *BRCA1/2* mutation carriers

**DOI:** 10.1186/s13058-015-0577-0

**Published:** 2015-05-19

**Authors:** Shandong Wu, Susan P Weinstein, Michael J DeLeo, Emily F Conant, Jinbo Chen, Susan M Domchek, Despina Kontos

**Affiliations:** 1 Department of Radiology, Hospital of the University of Pennsylvania, 1 Silverstein Building, 3400 Spruce Street, Philadelphia, PA 19104 USA; 2 Department of Biostatistics and Epidemiology, Perelman School of Medicine, University of Pennsylvania, 203 Blockley Hall, 423 Guardian Drive, Philadelphia, PA USA; 3 Department of Medicine, Hospital of the University of Pennsylvania, 3400 Civic Center Boulevard, 3 West Pavilion, Philadelphia, PA 19104 USA; 4 Present address: Imaging Research Division, Department of Radiology, University of Pittsburgh, 3362 Fifth Avenue, Pittsburgh, PA 15213 USA

## Abstract

**Introduction:**

We present a fully automated method for deriving quantitative measures of background parenchymal enhancement (BPE) from breast dynamic contrast-enhanced magnetic resonance imaging (DCE-MRI) and perform a preliminary evaluation of these measures to assess the effect of risk-reducing salpingo-oophorectomy (RRSO) in a cohort of breast cancer susceptibility gene 1/2 (*BRCA1/2*) mutation carriers.

**Methods:**

Breast DCE-MRI data from 50 *BRCA1/2* carriers were retrospectively analyzed in compliance with the Health Insurance Portability and Accountability Act and with institutional review board approval. Both the absolute (| |) and relative (%) measures of BPE and fibroglandular tissue (FGT) were computed from the MRI scans acquired before and after RRSO. These pre-RRSO and post-RRSO measures were compared using paired Student’s *t* test. The area under the curve (AUC) of the receiver operating characteristic (ROC) was used to evaluate the performance of relative changes in the BPE and FGT measures in predicting breast cancer that developed in these women after the RRSO surgery.

**Results:**

For the 44 women who did not develop breast cancer after RRSO, the absolute volume of BPE and FGT had a significant decrease (*P* < 0.05) post-RRSO, whereas for the 6 women who developed breast cancer, there were no significant changes in these measures. Higher values in all BPE and FGT measures were also observed post-RRSO for the women who developed breast cancer, compared with women who did not. Relative changes in BPE percentage were most predictive of women who developed breast cancer after RRSO (*P* < 0.05), whereas combining BPE percentage and |FGT| yielded an AUC of 0.80, higher than BPE percentage (AUC = 0.78) or |FGT| (AUC = 0.66) alone (both *P* > 0.02).

**Conclusions:**

Quantitative measures of BPE and FGT are different before and after RRSO, and their relative changes are associated with prediction of developing breast cancer, potentially indicative of women who are more susceptible to develop breast cancer after RRSO in *BRCA1/2* mutation carriers.

**Electronic supplementary material:**

The online version of this article (doi:10.1186/s13058-015-0577-0) contains supplementary material, which is available to authorized users.

## Introduction

Accurate risk assessment is critical for women at high risk of breast cancer to tailor risk reduction interventions [1]. High-risk women include those who have known genetic mutations (e.g., breast cancer susceptibility gene 1/2 [*BRCA1/2*]) or a strong family history of breast cancer [2]. In particular, women with *BRCA1/2* mutations are at a remarkably high lifetime risk, estimated to be up to 57 % for *BRCA1* carriers and 49 % for *BRCA2* carriers [3, 4]. Several risk reduction interventions are currently being used clinically, ranging from the less aggressive, such as lifestyle changes [5] and chemoprevention [6, 7], to those that are more aggressive, such as oophorectomy [8] and prophylactic mastectomy [9]. Although these interventions are effective in reducing cancer risk [10–12], they also have substantial side effects. For example, tamoxifen can induce endometrial cancer [13]; oophorectomy is associated with early menopause symptoms [14]; and prophylactic mastectomy is associated with morbidity and psychosocial effects [15].

A major current limitation is that it is uncertain what level of intervention is effective for individual women [16]. Given the lack of methods to weigh the benefit versus risks for different interventions, women decide based on their own preference and perception of risk rather than on an evidence-based approach. As a result, some women may be overtreated by undergoing unnecessary prophylactic mastectomies, whereas others may be undertreated and end up developing breast cancer. Risk-reducing salpingo-oophorectomy (RRSO) is a surgical procedure in which the ovaries and fallopian tubes are removed to help prevent ovarian and breast cancer in women. RRSO is recommended for *BRCA* mutation carriers (by the age of 35 to 40 years) and has been shown to reduce the risk of developing breast cancer by approximately 50 % [17, 18], with higher benefits associated with earlier age at surgery, and that of ovarian and/or fallopian tube cancer by approximately 80 % to 90 % [19]. Yet, there is no established standard to identify women most likely to benefit versus the ones who may need other aggressive interventions, such as prophylactic mastectomy. Therefore, methods are highly needed to better determine the likelihood of response to risk reduction interventions to better guide risk management decisions.

Dynamic contrast-enhanced magnetic resonance imaging (DCE-MRI) characterizes both the anatomic and physiologic activity of breast tissue [20]. Whereas mammographic density has been established through multiple studies as a risk factor for breast cancer [21, 22], mammography cannot differentiate the fibrous connective breast tissue from the hormonally responsive glandular tissue. In DCE-MRI, the glandular tissue enhances in the image as background parenchymal enhancement (BPE). Therefore, in addition to quantifying the overall fibroglandular tissue (FGT) content, MRI could provide a more sensitive biomarker of hormonal tissue activity by also quantifying BPE. Currently, MRI BPE and FGT are mainly estimated visually using the American College of Radiology Breast Imaging Reporting and Data System (BI-RADS) [23–25]. Although clinically useful, this kind of qualitative assessment is coarse, subjective, and has large inter- and intrareader variability [23], which makes it difficult to standardize. The purpose of this study was to present a fully automated method for deriving quantitative measures of BPE from breast DCE-MRI and perform a preliminary evaluation of these measures to assess the effect of RRSO in a cohort of *BRCA1/2* mutation carriers.

## Methods

### Dataset and imaging protocols

This retrospective study was compliant with the Health Insurance Portability and Accountability Act and approved by the University of Pennsylvania Abramson Cancer Center’s Clinical Trials Scientific Review and Monitoring Committee and Institutional Review Board with a waiver of consent granted, owing to the retrospective nature of the study and the minimal perceived risk to subjects. A cohort of 55 *BRCA1/2* mutation carriers was retrospectively identified. All women who underwent RRSO and had at least one pre-RRSO MRI and one post-RRSO MRI scan available during 1999–2011 at our institution were included in this study. Exclusion criteria were: (1) women with RRSO before their earliest MRI, (2) any prior history of radiated breast, and (3) bilateral mastectomy, if that was performed before undergoing at least one MRI after RRSO intervention. Women who had only pre-RRSO MRI scans or post-RRSO MRI scans, but not both, were also excluded. The age range of the study cohort was from 40 to 76 years, and the average was 51.8 years. Of these 55 women, 9 were diagnosed with breast cancer at a median of 4.8 years (range 1.8–13.3 years) of follow-up after RRSO. Each woman had a pre-RRSO DCE-MRI scan and a post-RRSO DCE-MRI scan available. Seventy-six percent (38/50) of the women were clinically pre-menopausal at the time of pre-RRSO imaging. Pre-RRSO MRI was performed ideally in the second week of the menstrual cycle in premenopausal women, as studies have shown that the least amount of BPE is seen in this time period [26, 27]. All women were clinically post-menopausal following RRSO. Two women (4 %) were receiving exogenous hormone replacement therapy before their pre-RRSO and post-RRSO scans. The first immediate MRI after RRSO is referred to herein as the post-RRSO MRI scan. The mean time to the post-RRSO MRI scan was 8.3 ± 7 months. Of the 55 women, 5 (including 3 women who developed breast cancer after RRSO) were excluded from our analysis because their DCE pre-contrast series in either the pre-RRSO or post-RRSO scanning were incomplete, so we were unable to quantitatively compute BPE. Therefore, a total of 100 DCE-MRI scans of 50 women (including 6 women who developed breast cancer after RRSO) were analyzed.

Women were scanned in the sagittal plane in the prone position in either a 1.5-T scanner (GE LX Echospeed, GE Healthcare, Nutley, NJ, USA, or Siemens Sonata, Siemens Medical Solutions, Malvern, PA, USA) or a 3-T scanner (Siemens Trio) using a dedicated surface breast coil array, including bilateral, fat-suppressed, T2-weighted (6530/86 [repetition time in milliseconds/echo time in milliseconds]) and slab interleaved, 3D, fat-suppressed, spoiled gradient echo sequences. The breast MRI scans of the study cohort were acquired with a range of parameters that evolved over the years (matrix size 512 × 512, 512 × 256, 256 × 256, or 192 × 192; slice thickness 2–4 mm; field of view 18–22 cm; flip angle 15° or 20°). Sequential post-contrast MRI series were acquired for approximately 6 min after contrast injection (Omniscan; GE Healthcare) per standard clinical protocol at our institution. A rapid bolus injection of 0.1 mmol/kg gadopentetate dimeglumine (Omniscan) followed by a 10-ml saline flush was administered in all women. The subtraction images (i.e., post-contrast minus pre-contrast; “SUB” for short) were routinely available for our analysis. MRI acquisition was fairly consistent in terms of the major aspects of the acquisition protocol, such as the MRI vendor, field strength, temporal resolution, and dynamic contrast enhancement protocol between the paired MRI scans of the same patient, whereas slight differences were introduced over time, owing to the technical evolution of the MRI protocol, mainly in matrix size (spatial resolution) and size of field of view as stated above, which was shown in previous studies not to have a major impact to our quantitative analysis [28, 29].

### Computerized quantification of background parenchymal enhancement

As an initial step, we used our previously validated fully automated method to segment and quantitatively measure FGT [28, 29]. Briefly, an edge extraction algorithm was first applied in the T1-weighted, non-fat-suppressed MRI scans to segment the breast from the image background and the chest wall [28], from which the absolute total breast volume (|Breast|) is estimated (in cubic centimeters). The fibroglandular tissue was then segmented within the breast volume using an atlas-aided fuzzy C-means algorithm [29]. The absolute volume (in cubic centimeters) of the segmented FGT (|FGT|) is computed, and FGT percentage was derived by using the following formula:

1 $$ \mathrm{F}\mathrm{G}\mathrm{T}\%=\frac{\left|\mathrm{F}\mathrm{G}\mathrm{T}\right|}{\left|\mathrm{Breast}\right|}\times 100 $$

Once FGT was segmented, we proceeded to compute BPE automatically based on the segmented FGT content. In our algorithm, BPE on the DCE-MR images was estimated by examining the relative difference of each voxel’s intensity (denoted by I in equation 2) in the SUB image relative to the intensity in the corresponding pre-contrast image. This voxel-wise enhancement ratio (R%) measures the relative intensity change as follows:

2 $$ \mathrm{R}\%=\left(\frac{{\mathrm{I}}_{\mathrm{post}}\hbox{-} \kern0.5em {\mathrm{I}}_{\mathrm{pre}}}{{\mathrm{I}}_{\mathrm{pre}}}\right)\times 100=\left(\frac{{\mathrm{I}}_{\mathrm{sub}}}{{\mathrm{I}}_{\mathrm{pre}}}\right)\times 100 $$

Because in principle only the FGT portion of the background parenchymal tissue should be enhance by the contrast agent uptake, we estimated the enhancement specifically over the FGT region. For this purpose, the FGT segmentation obtained in the T1-weighted, non-fat-suppressed images was translated to the DCE series images after applying a rigid registration step between the two scans [30]. Therefore, we could identify the enhancing voxels over the FGT region that had a value equal to or greater than a predefined enhancement ratio threshold R%_cutoff_, and BPE was computed as the total volume (in cubic centimeters) of these identified enhancing voxels:

3 $$ \mathrm{B}\mathrm{P}\mathrm{E}={\displaystyle {\sum}_{\mathrm{voxel}\in \mathrm{F}\mathrm{G}\mathrm{T}}\left(\mathrm{R}\%\ge \mathrm{R}{\%}_{\mathrm{Cutoff}}\right)} $$

We then estimated the absolute total volume of BPE (|BPE|) and derived the measure of BPE% as follows:

4 $$ \mathrm{B}\mathrm{P}\mathrm{E}\%=\frac{\left|\mathrm{B}\mathrm{P}\mathrm{E}\right|}{\left|\mathrm{B}\mathrm{reast}\right|}\times 100 $$

**Fig. 1:**
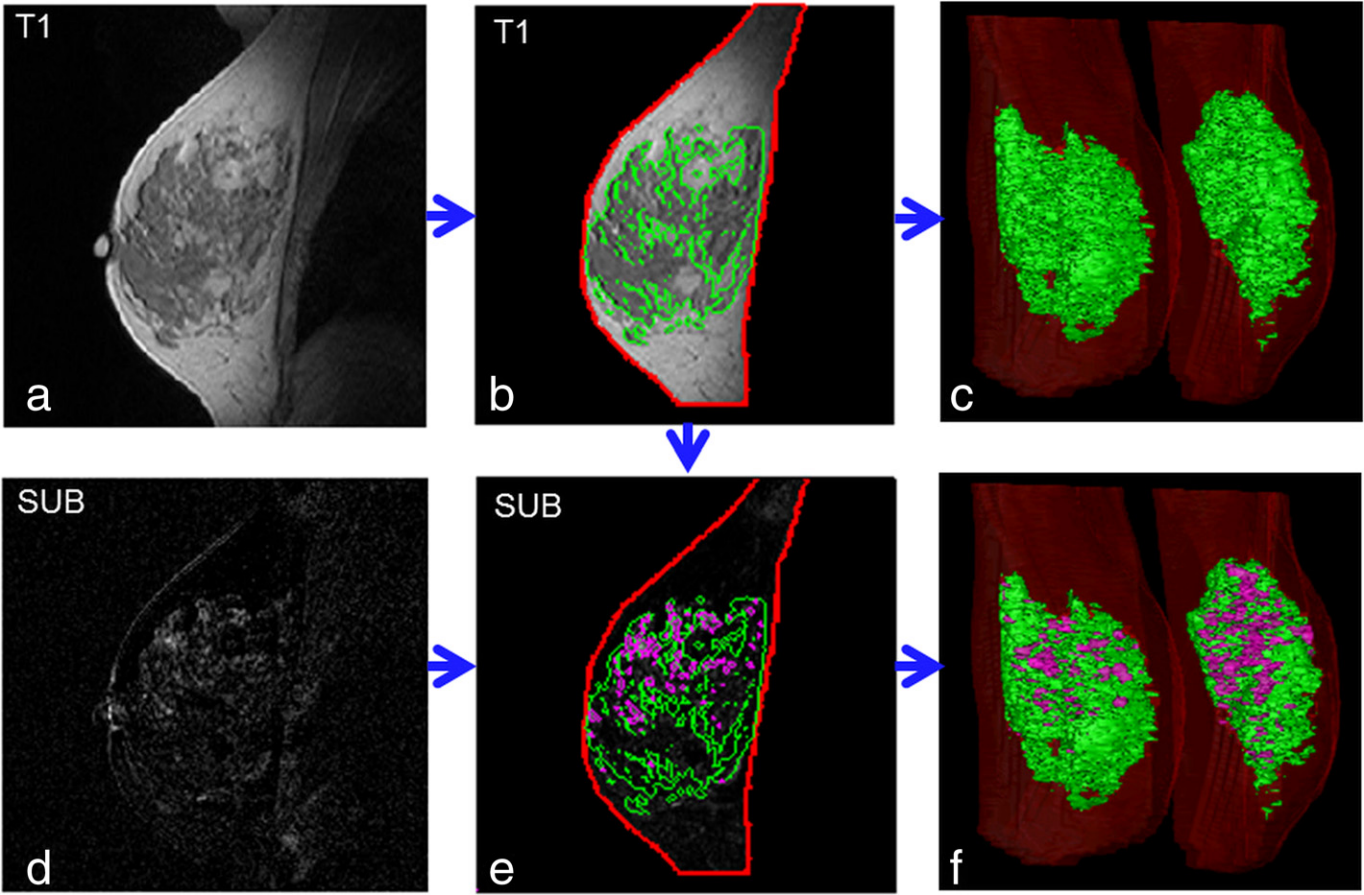
Illustration of our fully automated computer algorithm for quantifying magnetic resonance imaging (MRI) background parenchymal enhancement (BPE) and fibroglandular tissue (FGT). Representative 2D and 3D tissue segmentations in selected breast MRI scans are shown. **a** A breast MRI slice of the T1-weighted sequence. **b** Segmentation of the whole breast (*red contour*) and FGT (*green contour*). **c** 3D display of FGT (*green*) relative to the whole breast (*red*). **d** This dynamic contrast-enhanced MRI subtraction (SUB) image corresponds to the T1 slice shown in (a). **e** BPE (*purple contour*) estimated over the FGT area in the SUB image. **f** 3D display of BPE (*purple*), FGT (*green*), and whole breast (*red*)

A total of four quantitative measures were generated based on the above definitions: absolute and relative volumes of FGT, denoted as |FGT|, FGT%, respectively; and absolute and relative volumes of BPE, denoted as |BPE| and BPE%, respectively (Fig. 1).

In typical clinical protocols, there may be multiple post-contrast time points and SUB images available [20]. Multiple post-contrast images acquired at different time points reflect changes in signal intensity induced by the uptake of the contrast agent over time [31, 32]. Therefore, in our analysis, we evaluated the BPE estimated using the first and third time point SUB images for the purpose of comparing the effect that early versus delayed tissue enhancement may have on the estimation of BPE (i.e., SUB 1 vs. SUB 3).

### Statistical analysis

As there is currently no established value for the R%_cutoff_, we estimated BPE using values for the R%_cutoff_ ranging from 0 % to 100 % with increments of 10 %. For each of the FGT and BPE measures, the mean (±SD) was compared between the pre- and post-RRSO MRI scans for the 44 women who did not develop breast cancer post-RRSO versus the 6 women who did, using a two-sided Student’s paired *t* test at a significance level of 0.05. To jointly test differences in contrasting the two independent variables (i.e., developing vs. not developing breast cancer and pre-RRSO status vs. post-RRSO status), analysis of variance (ANOVA) was also performed. Given the preliminary nature of our study, we also used the false discovery rate (FDR), a family-wise approach, to correct for multiple comparisons. In addition, the relative difference (i.e., [post-RRSO − pre-RRSO]/pre-RRSO) in each FGT and BPE measure was computed for all women, and these relative differences were evaluated for their association with the odds of developing breast cancer post-RRSO using logistic regression. Receiver operating characteristic (ROC) curves were generated, and the area under the curve (AUC) was derived to evaluate predictive value in distinguishing women who developed breast cancer after RRSO versus the ones who did not. ROC curves were generated by using developing versus not developing cancer post-RRSO as a binary ground truth label and the output (i.e., the predicted probability of cancer) generated from the fitted logistic regression model as the decision variable. DeLong’s test was used to compare the different AUCs. As a preliminary evaluation, we also sought to determine if an optimal value for R%_cutoff_ exists that generates measures most predictive of BPE. Toward this end, we also used leave-one-out cross-validation (LOOCV) to compare AUCs computed based on relative changes of BPE between post-RRSO scans and pre-RRSO scans, derived across the same range of the R%_cutoff_ values (from 0 % to 100 %; see Additional file 1).

## Results

**Fig. 2:**
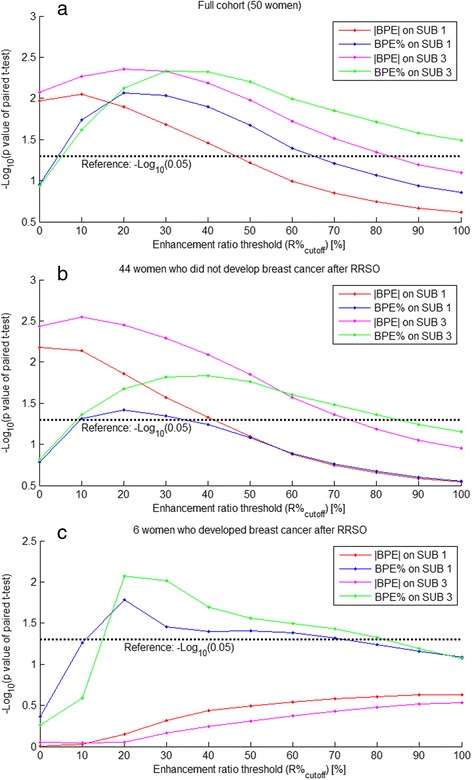
Manhattan plot of the *P* value profiles of paired Student’s *t* test comparing the pre-RRSO and post-RRSO measures of background parenchymal enhancement (BPE) (both |BPE| and BPE %) computed by using a range of values (from 0 % to 100 %) for the R %_cutoff_, indicating a range of the R %_cutoff_ values on which the difference between the pre-RRSO and post-RRSO BPE measures become significant (*P* < 0.05). RRSO, Risk-reducing salpingo-oophorectomy. **a** Results for the full cohort (i.e., a total of 50 women). **b** Results for the 44 women who did not develop breast cancer post-RRSO. **c** Results for the 6 women who developed breast cancer post-RRSO. *Abbreviation*: SUB, Subtraction image (i.e., post-contrast minus pre-contrast)

The *P* values derived from the *t* test comparing the BPE-related measures in the pre-RRSO scans versus the post-RRSO scans for a range of values of the R%_cutoff_ are shown in Manhattan plots in Fig. 2. As can be seen from the *P* value profiles of the full cohort (Fig. 2a), when using SUB 1 to estimate BPE, there was a consistently significant (*P* < 0.05) decrease after the RRSO for |BPE| when the R%_cutoff_ fell within the range from 0 % to 40 %, and a similar observation held true for BPE% when the R%_cutoff_ was in the range of 10 % to 60 %. Likewise, when using SUB 3, there was a significant decrease of the BPE measures after RRSO; however, the corresponding ranges of the R%_cutoff_ where this decrease was significant expanded to an overall wider range of 0 % to 80 % for |BPE| as well as from 10 % to 100 % for BPE%. When we examined separately the women who did not develop breast cancer post-RRSO, we observed similar trends (Fig. 2b). However, there were no significant changes in |BPE| for women who developed breast cancer post-RRSO, regardless of whether SUB 1 or SUB 3 was used for the |BPE| estimation and across the entire range of the R%_cutoff_ (Fig. 2c).

**Fig. 3:**
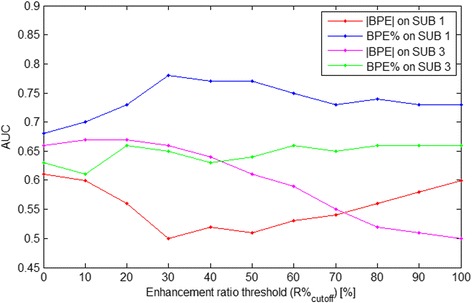
The areas under the receiver operating characteristic curves (AUCs) of the predictive performance using relative changes in the different background parenchymal enhancement (BPE) measures across the entire range of R %_cutoff_ values were employed to predict women who developed breast cancer post-RRSO, where BPE % estimated from subtraction image 1 (i.e., post-contrast minus pre-contrast [SUB 1]) shows the highest predictive value (AUC 0.78, *P* = 0.09) for an R %_cutoff_ of 30 %

The ROC AUCs for predicting the women who developed breast cancer post-RRSO based on the relative differences of their BPE measures before and after RRSO are shown in Fig. 3, with BPE% estimated from SUB 1 having the most predictive value (AUC 0.78, *P* = 0.09) for an R%_cutoff_ of 30 %. This cutoff of 30 % appeared to be consistent and was further confirmed by the LOOCV-generated AUCs (see Additional file 1: Figure S1).

**Fig. 4:**
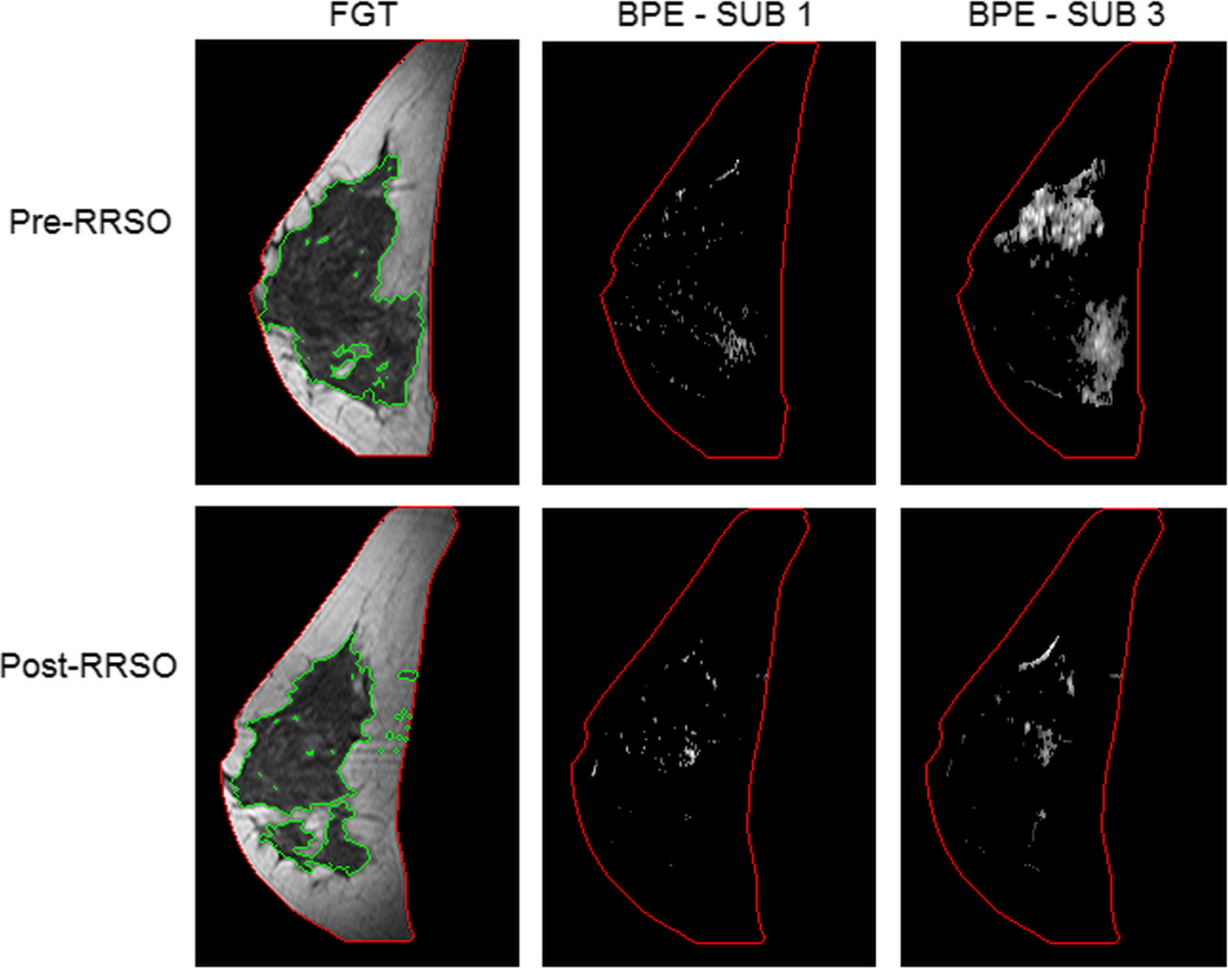
Representative examples of fibroglandular tissue (FGT) and background parenchymal enhancement (BPE) from a magnetic resonance imaging (MRI) slice obtained pre-RRSO and post-RRSO in a woman who did not develop breast cancer after undergoing risk-reducing salpingo-oophorectomy (RRSO). FGT is circumscribed by *green contours*. BPE is estimated in both the first and third subtraction series (SUB 1 and SUB 3, respectively). This 40-year-old (at time of RRSO) woman had her pre-RRSO MRI at 6 months before RRSO and her post-RRSO MRI at 1 month after RRSO. She had no personal history of breast cancer or other cancer and had no breast cancer diagnosis for up to 9 years of post-RRSO follow-up. This example illustrates that there was a decrease of BPE and FGT after her RRSO. The volumetric pre-RRSO BPE % values were 1.5 % (SUB 1) and 6.5 % (SUB 3) and after RRSO, and BPE % values were 0.6 % (SUB 1) and 3.6 % (SUB 3)

**Fig. 5:**
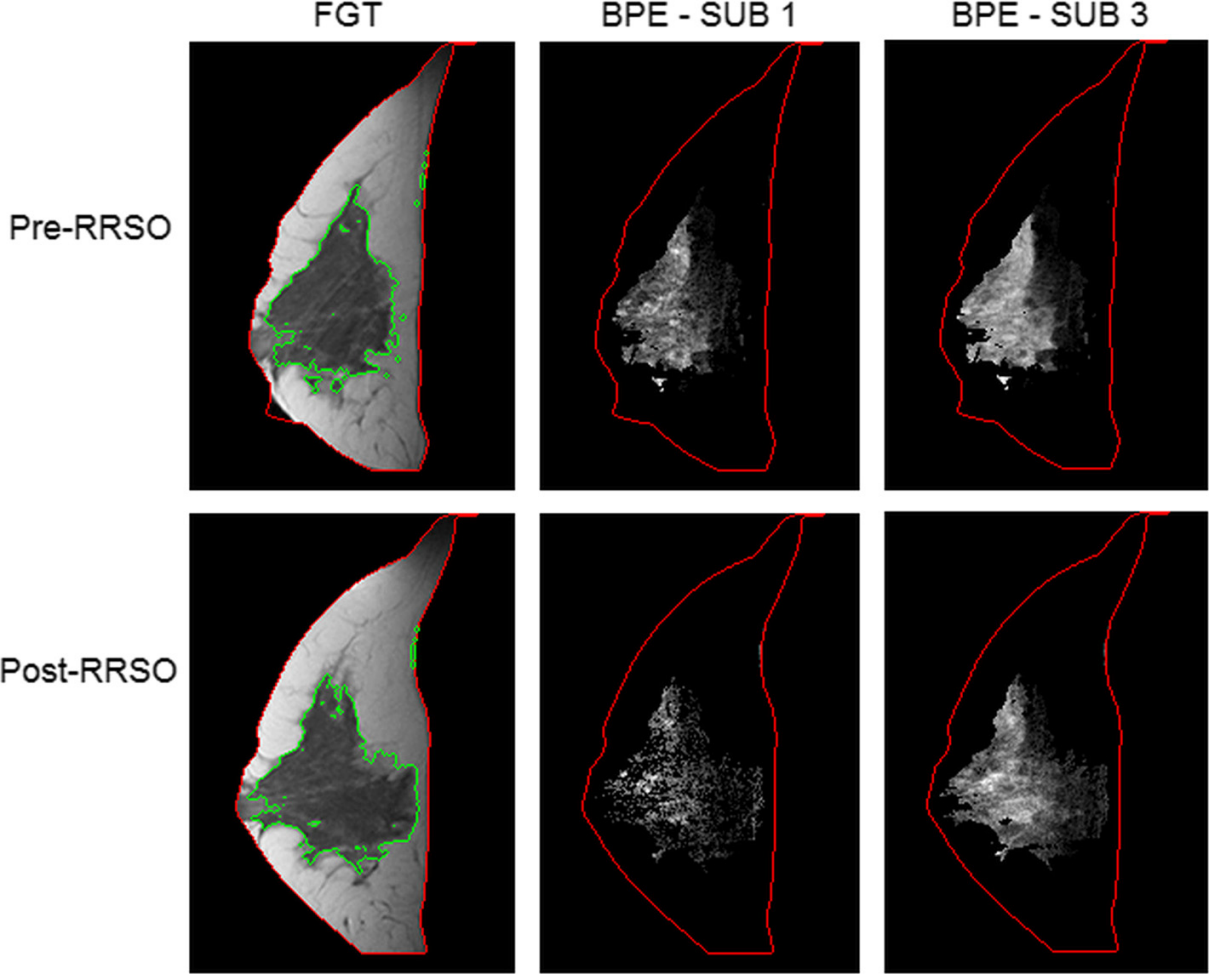
Representative examples of fibroglandular tissue (FGT) and background parenchymal enhancement (BPE) from a magnetic resonance imaging (MRI) slice obtained pre-RRSO and post-RRSO in a woman who developed breast cancer after undergoing risk-reducing salpingo-oophorectomy (RRSO). FGT is circumscribed by *green contours*. BPE is estimated in both the first and third subtraction series (SUB 1 and SUB 3, respectively). This 36-year-old (at time of RRSO) woman had her pre-RRSO MRI at 1 month before RRSO and her post-RRSO MRI at 6 months after RRSO. Breast cancer (ductal carcinoma in situ) was diagnosed in her right breast 6 years after RRSO, and her final pathologic examination showed in situ carcinoma with 2 foci of microinvasion (<0.1 cm). This example shows no decrease of BPE and FGT after RRSO. The volumetric pre-RRSO BPE % values were 10.7 % (SUB 1) and 10.6 % (SUB 3) and after RRSO, and BPE % values were 7.1 % (SUB 1) and 8.5 % (SUB 3)

For this representative R%_cutoff_ of 30 %, the values of all MRI measures, including the BPE measures computed in both SUB 1 and SUB 3, are shown in Table 1. In terms of the FDR-corrected *P* values, for the subcohort of 44 women who did not develop breast cancer post-RRSO, BPE measures computed in SUB 3, as well as |FGT|, had a significant decrease (*P* < 0.05) post-RRSO. For the six women who developed breast cancer post-RRSO, all FGT (|FGT| and FGT%) and BPE (|BPE| and BPE%) measures did not show significant changes post-RRSO, except for a significant decrease in BPE% computed in SUB 3. In terms of the ANOVA, BPE% SUB1 stood out as the most statistically significant measure when we jointly contrasted the pre-/post-RRSO status and developing versus not developing cancer groups. The women who developed breast cancer had higher average values in all the BPE and FGT measures post-RRSO than the women who did not (*P* = 0.046). Examples of FGT and BPE pre-RRSO and post-RRSO are shown in Figs. 4 and 5 for selected patients in each subgroup.

**Table 1:** Comparison of the fibroglandular tissue and background parenchymal enhancement measures before and after risk-reducing salpingo-oophorectomy

		|FGT| [cm^3^]	FGT % [ %]	|BPE| SUB 1 [cm^3^]	BPE % SUB 1 [ %]	|BPE| SUB 3 [cm^3^]	BPE % SUB 3 [ %]
44 women who did not develop breast cancer post-RRSO	Pre-RRSO	83.2 ± 63.8	11.3 ± 8.1	28.2 ± 44.2	3.8 ± 4.5	43.0 ± 49.7	6.0 ± 5.9
	Post-RRSO	60.4 ± 50.1	10.3 ± 7.5	12.2 ± 11.8	2.3 ± 1.8	21.0 ± 15.2	3.9 ± 2.5
	*P* value	0.005	0.23	0.027	0.045	0.005	0.015
	FDR-corrected *P* value	0.03	0.34	0.06	0.078	0.03	0.046
6 women who developed breast cancer post-RRSO	Pre-RRSO	65.1 ± 60.2	11.6 ± 3.8	26.7 ± 12.6	7.3 ± 4.6	40.0 ± 31.3	8.2 ± 3.8
	Post-RRSO	72.9 ± 38.2	11.2 ± 7.6	20.5 ± 19.2	3.3 ± 2.3	33.6 ± 20.1	5.4 ± 3.2
	*P* value	0.78	0.83	0.48	0.035	0.68	0.0096
	FDR-corrected *P* value	0.83	0.83	0.64	0.070	0.82	0.038
ANOVA *P*-value		0.83	0.24	0.058	0.0057	0.021	0.023
FDR-corrected ANOVA *P*-value		0.83	0.29	0.087	0.034	0.046	0.046
AUC		0.66	0.57	0.48	0.78	0.66	0.65

Data are presented as mean ± SD, where background parenchymal enhancement (BPE) is estimated using the representative R %_cutoff_ of 30 % for subtraction images 1 and 3 (i.e., post-contrast minus pre-contrast; SUB 1 and SUB 3, respectively). *P* values were derived by paired Student’s *t* test comparing pre-RRSO and post-RRSO measures for each group. In analysis of variance, the *P* values represent our jointly contrasting the groups developing vs. not developing cancer and pre-RRSO vs. post-RRSO status as the two independent variables. False discovery rate (FDR)-corrected *P* values are also provided as a means to adjust for multiple comparisons. AUC refers to the area under the receiver operating characteristic curve for the logistic regression models to predict women who developed breast cancer after RRSO, based on the relative differences (i.e., [post-RRSO − pre-RRSO]/pre-RRSO) in each measure. *Abbreviation*: RRSO, Risk-reducing salpingo-oophorectomy

**Fig. 6:**
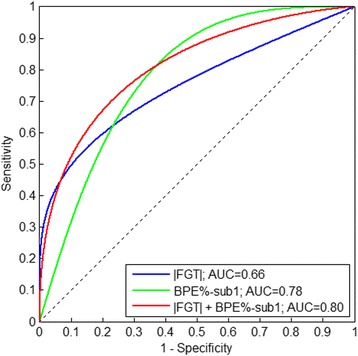
Area under the receiver operating characteristic curve (AUC) for the logistic regression models to predict women who developed breast cancer after risk-reducing salpingo-oophorectomy (RRSO), based on combination of relative changes (i.e., [post-RRSO − pre-RRSO]/pre-RRSO) of their absolute fibroglandular tissue (|FGT|) and background parenchymal enhancement first subtraction series (BPE % SUB 1) measures (i.e., best in Table 1). Combining BPE % and |FGT| yielded a higher AUC of 0.80 than that of BPE % (AUC 0.78) or |FGT| (AUC 0.66) alone (both *P* > 0.2)

Finally, for the R%_cutoff_ of 30 %, we also tested the combination of the most predictive measures of FGT and BPE (i.e., |FGT| and BPE% SUB 1) as joint predictors in a multivariable logistic regression model, which yielded an improved AUC of 0.80 (*P* = 0.08 for |FGT| and *P* = 0.09 for BPE% SUB 1), but the AUCs were not statistically significantly higher (*P* > 0.2) than the AUC of either the BPE% SUB 1 (AUC 0.78, *P* = 0.09) or |FGT| (AUC 0.66, *P* = 0.03) alone (Fig. 6). The Spearman correlation between the |FGT| and BPE% SUB 1 was 0.09 (p = 0.5).

**Fig. 7:**
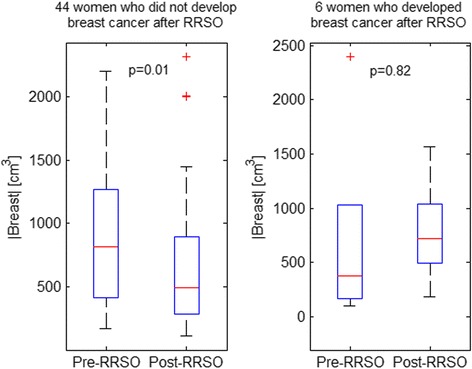
Whole-breast volume (|Breast|) changes after risk-reducing salpingo-oophorectomy (RRSO) showing a significant decrease for the 44 women who did not develop breast cancer post-RRSO but a non-significant increase for the 6 women who developed breast cancer after RRSO. The changes may be due to overall changes in the breast disuse, including changes in the fat content, induced by hormonal changes due to the RRSO surgery

Whereas there was no significant decrease in |BPE| for the women who developed breast cancer after RRSO, there was a significant change in BPE%. This was an interesting observation, which we investigated further by also looking into potential changes of the total breast volume. We found that the total breast volume (|Breast|) also changed after RRSO (Fig. 7). Specifically, whereas the total breast volume after RRSO also decreased for the women who did not develop breast cancer (*P* = 0.01), our preliminary analysis suggested that the total volume had no significant change for the women who developed breast cancer (*P* = 0.82).

## Discussion

In our study, quantitative measures of BPE and FGT were computed with data derived from breast DCE-MRI using a fully automated computerized method, and they were subsequently evaluated for a pilot cohort of *BRCA1/2* mutation carriers to assess the effect of RRSO. Although this is a preliminary study with a small discovery dataset requiring further validation in a larger cohort, overall our results indicate that these measures could be useful for assessing response to RRSO and potentially identifying the women who may still be susceptible to developing breast cancer after their surgery. In terms of the *t* test *P* values shown in Table 1, the absolute volume of BPE and FGT appeared to have a significant decrease post-RRSO for women who did not develop breast cancer (with a median of 4.8 years of follow-up), whereas there were no significant changes in these measures for women who developed breast cancer post-RRSO. The 6 women who developed breast cancer had higher average values for all the BPE and FGT measures post-RRSO compared with the 44 women who did not. It is also interesting to note that FGT% and |BPE| (both on SUB 1 and SUB 3) started off with similar average values before RRSO in both groups and that the significant decrease of |BPE| was observed only in the group of the 44 women who did not develop breast cancer post-RRSO. In the evaluation of the relative changes of these MRI measures in predicting breast cancer development post-RRSO, it appeared that the combination of BPE% SUB1 and |FGT| provided the highest prediction accuracy—higher than either of them alone. The low correlation between these measures suggested that they may be independently associated to breast cancer, although, owing to the marginal statistical significance, larger studies with sufficient sample size are warranted to further validate this claim. Overall, our results indicated that BPE% SUB1 was the most significant measure in predicting breast cancer after RRSO, and specifically that the percentage measures of BPE, particularly quantified in the first post-contrast SUB sequence (i.e., BPE% SUB 1), may be most relevant as an imaging biomarker for assessing the effect of RRSO.

Our observations are in accordance with previously reported reader studies [24, 33] in which the 4-scale qualitative BI-RADS assessment was used to evaluate changes in BPE and FGT between pre-RRSO and post-RRSO MRI scans on *BRCA1/2* mutation carriers. In their study of 55 patients, DeLeo et al. [33] reported a significant reduction in BPE but no significant change in FGT. In their study of 18 patients, Price et al. [24] observed significant decreases in both BPE and FGT after RRSO and that BPE had a greater extent than FGT in the observed decrease. In addition, no previous study researchers have evaluated the changes in BPE and FGT measures between pre-RRSO and post-RRSO scans for predicting the development of breast cancer post-RRSO, which is an important clinical endpoint of interest. In the reader studies [24, 33], only a fair to moderate interreader agreement (κ = 0.3–0.6) was reported for the qualitative assessment of BPE. On the contrary, fully automated computerized methods, such as the ones evaluated in our study, can provide objective and reproducible quantitative measures that alleviate the subjectivity of intra- and inter reader variability in the qualitative MRI assessment. In addition, fully automated assessment is more time-efficient: Automated quantification of FGT and BPE in our study took approximately a total of 5 min per scan, compared, for example, with manual segmentation of FGT previously reported at about 55 min per MRI scan [29]. As such, automated methods are critical to accelerating the translation of these imaging measures into routine clinical application. In recent studies, researchers have also reported that higher measures of BPE and FGT may indicate a higher risk of developing breast cancer [23]. Along these lines, our results suggest that our measures could be used to determine which women may be more likely to develop breast cancer after RRSO and could potentially benefit from alternative risk reduction interventions such as prophylactic mastectomy.

We also compared the effects of BPE quantified on SUB 1 versus SUB 3. Whereas we observed similar trends in changes for both |BPE| and BPE% (Fig. 2), there was a difference in the range of the R%_cutoff_ at which these changes became significant (*P* < 0.05). The larger range observed in SUB 3 may reflect that there is more uptake of contrast agent in the breast tissue owing to a longer uptake time; likewise, this may also explain the smaller range observed in SUB 1, on which the amount of contrast enhancement was less owing to a shorter uptake time. At the same time, we observed that the BPE% derived from SUB 1 was the most predictive measure among all BPE measures evaluated. Therefore, determining whether measures from the early-stage or, alternatively, delayed-stage post-contrast series are more predictive of a response to RRSO requires further validation.

As ovaries are removed, RRSO results in great reduction in the amount of the circulation of the endogenous hormones estrogen and progesterone. As a result of this effect, all 50 women essentially became clinically post-menopausal after RRSO. Studies have shown that BPE and FGT decrease after menopause in great proportions of healthy women [34] and that BPE increases in post-menopausal women who have had recent hormone replacement therapy [35]. Therefore, this may explain the post-RRSO changes of BPE and FGT observed in our study as an effect of the reduction in the corresponding endogenous reproductive hormone levels. As for the total breast volume changes, whereas the biological basis of the observation cannot be interpreted solely on the basis of our study, we speculate that it may be due to overall changes in the breast disuse, including changes in the fat content, induced by hormonal changes resulting from the RRSO surgery.

Limitations of our study must also be noted. In this pilot study, we used a relatively small retrospective cohort in which we had only a few women who developed breast cancer post-RRSO, which therefore limited our power to derive conclusive interpretations of the related findings. With such a small dataset, the *P* values should be assessed with caution. That being said, with this preliminary evaluation, mainly for hypothesis generation, we believe our results will be instrumental in guiding the design and appropriate power calculation for a larger prospective study. In addition, in this exploratory study, our initial investigation was focused on the main effect analysis of RRSO on BPE. To investigate the direct effect of RRSO, it will be important in future studies to adjust for factors such as exogenous hormonal treatment, medications, diet, and other possible confounding factors. In addition, as no established R%_cutoff_ currently exists, we examined the effects of BPE using a range for R%_cutoff_ from 0 % up to 100 %, which may have introduced a bias via multiple comparisons. Given the preliminary nature of our study mainly for generating hypotheses, we used a family-wise approach (i.e., FDR) because it was more appropriate to adjust our significance threshold for multiple comparisons. In our preliminary evaluation, we found that there is a range of values for R%_cutoff_ in which all these measures were able to achieve statistical significance (Fig. 2) and that a 30 % R%_cutoff_ had the best predictive performance for our study population. On the basis of the promising performance of our method, further work is warranted to fully optimize algorithm parameters and validate our findings in larger populations. Finally, these computer algorithms require additional evaluation to determine generalizability to a range of different MRI protocols and scanners.

## Conclusions

Quantitative measures of BPE and FGT were computed using data derived from breast DCE-MRI scans using a fully automated computerized method and were subsequently evaluated for a pilot cohort of *BRCA1/2* mutation carriers for assessing the effect of RRSO. The relative changes of the BPE and FGT between post-RRSO and pre-RRSO are associated with predicting development of breast cancer after RRSO. Our results suggest that these measures may be useful for predicting response to RRSO and ultimately assisting in clinical decision making regarding risk-reducing interventions for *BRCA1/2* carriers. We envision a setting in which individualized risk assessment and patient education are combined to empower women with knowledge about their personal risk while providing much-needed quantitative tools for clinicians. As it is currently recommended that high-risk women also undergo annual MRI screening [2], there is an opportunity to use novel imaging approaches to measure breast tissue properties related to an individual woman’s response to risk reduction interventions. Our findings, after further validation in larger prospective studies, could be translated in the clinic to develop an automated tool for integrated breast MRI assessment and RRSO response prediction to aid clinical decision making. As such, our proposed imaging biomarkers could result in a more effective, evidence-based approach for risk reduction management of high-risk women using measures of individualized response to each intervention. Such a personalized approach for tailored risk reduction could improve risk management, quality of life, and ultimately outcomes for women at high risk for breast cancer.

## Additional file

Additional file 1: Figure S1. Determination of an optimal parameter value for BPE quantification using leave-one-out cross validation. This supplemental material describes what value is identified by using leave-one-out cross-validation as an optimal threshold for parameter R%_cutoff_ for quantifying BPE.

## Abbreviations

ANOVA: Analysis of variance
AUC: Area under the curve
BI-RADS: Breast Imaging Reporting and Data System
BPE: Background parenchymal enhancement
*BRCA1/2*: Breast cancer susceptibility gene 1/2
DCE-MRI: Dynamic contrast-enhanced magnetic resonance imaging
FDR: False discovery rate
FGT: Fibroglandular tissue
LOOCV: leave-one-out cross-validation
ROC: Receiver operating characteristic
RRSO: Risk-reducing salpingo-oophorectomy
SUB: Subtraction
