# Erratum to: Quantitative assessment of background parenchymal enhancement in breast MRI predicts response to risk-reducing salpingo-oophorectomy: preliminary evaluation in a cohort of *BRCA1/2* mutation carriers

**DOI:** 10.1186/s13058-015-0651-7

**Published:** 2015-11-24

**Authors:** Shandong Wu, Susan P. Weinstein, Michael J. DeLeo, Emily F. Conant, Jinbo Chen, Susan M. Domchek, Despina Kontos

**Affiliations:** Department of Radiology, Hospital of the University of Pennsylvania, 1 Silverstein Building, 3400 Spruce Street, Philadelphia, 19104 PA USA; Department of Biostatistics and Epidemiology, Perelman School of Medicine, University of Pennsylvania, 203 Blockley Hall, 423 Guardian Drive, Philadelphia, PA USA; Department of Medicine, Hospital of the University of Pennsylvania, 3400 Civic Center Boulevard, 3 West Pavilion, Philadelphia, 19104 PA USA; Present address: Imaging Research Division, Department of Radiology, University of Pittsburgh, 3362 Fifth Avenue, Pittsburgh, 15213 PA USA

## Erratum

With this erratum, we would like to clarify key differences between our current publication [1], and prior work published in the American Journal of Roentgenology (AJR) and cited as reference [33] (reference [2] here), from which our current study includes a subset of the patient population. First, this prior work was a qualitative reader study, in which fibroglandular tissue (FGT) and background parenchymal enhancement (BPE) were evaluated subjectively using visual assessment by two clinical readers into the four qualitative Breast Imaging Reporting and Data System (BI-RADS) categories. In contrast, in our study published in Breast Cancer Research (BCR), we performed quantitative analysis, using a fully-automated computerized method developed by our group, reported for the first time for quantifying BPE in breast MRI, where a key aspect for BPE quantification, the optimal evaluation and determination for the parameter of enhancement ratio (R%), was also investigated in association to patient cancer outcomes. In addition to evaluating group-wise changes in the means of FGT and BPE between pre- and post- risk-reducing salpingo-oophorectomy (RRSO), we also included a comparison of the effect for BPE assessed in three sequential post-contrast sequences acquired at different time-points, and most importantly conducted an additional evaluation of predicting development of breast cancer after RRSO, using the quantitative changes of the FGT and BPE measures post RRSO, which was not evaluated in the AJR study. This is a key investigation, as predicting subsequent breast cancer is the main outcome of interest for improving clinical-decision making for risk-evaluation and risk-reduction management of women with BRCA mutations. The quantitative analysis reported in our study published in BCR has shown overall superior performance to the prior qualitative study published in AJR, providing a potential precision-medicine approach for the quantification of MRI BPE as a biomarker for guiding risk-reduction management in women at high-risk for breast cancer.
