# Supplementary material for: Quantitative assessment of background parenchymal enhancement in breast MRI predicts response to risk-reducing salpingo-oophorectomy: preliminary evaluation in a cohort of BRCA1/2 mutation carriers
Source: Breast Cancer Res. 2015 May 19;17:67. doi: 10.1186/s13058-015-0577-0 (PMC4481125; doi:10.1186/s13058-015-0577-0)
Supplement: Additional file 1: Figure S1. — Determination of an optimal parameter value for BPE quantification using leave-one-out cross validation. This supplemental material describes what value is identified by using leave-one-out cross-validation as an optimal threshold for parameter R%cutoff for quantifying BPE. [file 13058_2015_577_MOESM1_ESM.pdf]

Determination of an optimal parameter value for BPE quantification using leave-one-out cross validation

We have tested the leave-one-out cross validation (LOOCV) to determine optimal cut-off value for parameter $R\%_{cutoff}$, in which AUCs were computed based on LOOCV using before and after-RRSO relative BPE changes, derived across the same range of the $R\%_{cutoff}$ values (from 0% to 100%). Based on the most significant measure (i.e., BPE%, according to the ANOVA test in Table 1), we again found that $R\%_{cutoff}$ =30% yielded the highest cross-validated AUC (see below Figure S1). This finding of $R\%_{cutoff}$=30% is consistent with what we have observed from the analyses using the whole dataset, as reported in Figure 3.

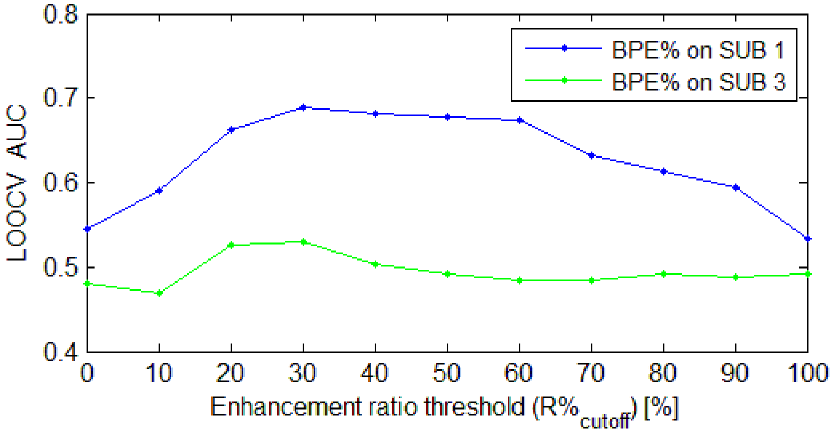

Figure S1: The ROC AUCs (based on the leave-one-out cross validation) of the predictive performance when using relative changes in the different BPE% measures across the entire range of $R\%_{cutoff}$ values to predict women who developed breast cancer post-RRSO.
